# Breast cancer cell resistance to hormonal and targeted therapeutics is correlated with the inactivation of the NR6A1 axis

**DOI:** 10.20517/cdr.2024.69

**Published:** 2024-11-23

**Authors:** Olga E. Andreeva, Danila V. Sorokin, Svetlana V. Vinokurova, Pavel B. Kopnin, Nadezhda V. Elkina, Alexey N. Katargin, Radik S. Faskhutdinov, Diana I. Salnikova, Alexander M. Scherbakov, Mikhail A. Krasil’nikov

**Affiliations:** ^1^N.N. Blokhin National Medical Research Center of Oncology, the Ministry of Health of Russia, Moscow 115522, Russia.; ^2^Gause Institute of New Antibiotics, Moscow 119021, Russia.; **^#^** Authors contributed equally.

**Keywords:** Breast cancer, tamoxifen, rapamycin, methylation, DNMT3A, *NR6A1*, Snail, estrogen receptor α, MCF-7 cells

## Abstract

**Aim:** Resistance to hormonal and targeted therapies in breast cancer limits treatment efficacy. Epigenetic alterations, including changes mediated by DNA methyltransferases, play a key role in this process. Previously, we identified that resistance to tamoxifen and rapamycin is associated with the suppression of DNMT3A. This study aims to further explore the mechanisms underlying this suppression, with a focus on identifying NR6A1 as a novel regulatory factor.

**Methods:** Acquisition of resistant breast cancer cell sublines, MTT-test, immunoblotting, transient transfection and reporter analysis, lentiviral infection, qRT-PCR, and analysis of methylation using bisulfite pyrosequencing.

**Results:** Our findings indicate that the development of cross-resistance in breast cancer cells to hormonal and targeted therapies involves a shift in cell signaling to alternative AKT pathways, marked by a localized suppression of the NR6A1/DNMT3A axis and associated DNA methylation changes. We demonstrated the critical role of NR6A1 downregulation in resistance development. Additionally, we observed activation of Snail - a key regulator in the epithelial-mesenchymal transition - as a mediator of the effects of NR6A1 depletion, establishing a direct link between Snail expression and resistance formation.

**Conclusion:** The coordinated suppression of NR6A1 and DNMT3A may contribute to sustaining the resistant phenotype in breast cancer cells. This pathway could serve as a predictive marker, helping guide the selection of optimal therapeutic strategies for breast cancer treatment in the future.

## INTRODUCTION

The recent active development of targeted and hormonal therapy has resulted in a considerable prolongation of the lifespan of cancer patients, but the effectiveness of therapy is largely limited by the development of tumor resistance to applied drugs, both pre-existing (*de novo*) and acquired during treatment^[1-4]^. The features of resistance development to hormone therapy^[5,6]^ are disruption of the steroid receptor machinery, including a decrease in the content and/or activity of receptors, ligand-independent activation of receptors, and disruption of the balance between activator proteins and receptor suppressors. Similarly, resistance to targeted drugs is associated with the modifications of direct target proteins as well as the rearrangement of respective signaling pathways^[7]^. However, as in the case of hormone resistance as well as in the progression of resistance to targeted drugs, the necessary and key factor in the formation of resistance is the activation of parallel signaling pathways that respond to proliferative signals by bypassing the blocked pathways^[8-11]^. Such rearrangement may be associated with the accumulation of activating mutations and/or reactivation of epigenomic factors that modulate the signaling pathways of resistant cells. Among the factors of epigenomic regulation, the most important role belongs to DNA methylation enzymes participating in the regulation of the expression of key genes in cancer cells.

The role of methylation of DNA in the promotion of cancer cell resistance is well known; driver genes whose methylation or demethylation is correlated with the formation of drug resistance have been described and identified, including genes encoding ABC transporters, receptors, growth factors, DNA repair enzymes, *etc*.^[11,12]^ Less is understood about the regulation of DNMTs in breast cancer cells during resistance development. DNMT3A and DNMT1 have been found to be expressed in breast cancer cells; some non-coding RNAs have been identified as regulators of DNMTs^[13-15]^, but to a large extent, the involvement of DNMTs in resistance development remains an open question.

Earlier, we described the advancement of cross-resistance of *in vitro* cultured breast cancer cells to hormonal agents (antiestrogen tamoxifen) and targeted drugs (mTOR inhibitors rapamycin and metformin). We have shown that, in particular, cell cross-resistance is based on the activation of a major proliferative and anti-apoptotic pathway, PI3K/AKT signaling. We have shown for the first time that the development of cross-resistance is linked to reduced expression of DNA methyltransferase 3A (DNMT3A) and demonstrated a clear link between DNMT3A suppression and the development of hormonal or target resistance^[16,17]^.

Recently, one of the transcriptional repressors, NR6A1 (Nuclear Receptor Subfamily 6 Group A Member 1, otherwise known as GCNF (Germ Cell Nuclear Factor)), was described as one of the proteins responsible for the control of DNMTs^[18,19]^. NR6A1 is a member of the nuclear receptor superfamily of ligand-activated transcription factors and can bind to conservative DNA sequences, acting as a transcriptional repressor. NR6A1 is very lowly expressed in the mammary glands but highly expressed in some types of breast cancer^[20,21]^; the ability of NR6A1 to interact with other types of co-repressors such as ERR, N-CoR, and SMRT was demonstrated in various experimental models^[22,23]^. With that, NR6A1 was found to form active complexes with various types of DNMTs, resulting in the recruitment of the latter to specific DNA sequences and subsequently modulating methylation patterns^[24-26]^.

Here, we showed that the promotion of cross-resistance of breast tumor cells to hormonal and targeted drugs is underpinned by a switch of cell signaling to unblocked AKT pathways associated with a focal suppression of the NR6A1/DNMT3A axis and the corresponding alterations in DNA methylation, and illustrated the significance of NR6A1 decrease in the development of cell resistance. Additionally, we have described the phenomenon of the activation of Snail, one of the pivotal proteins involved in the epithelial-mesenchymal transition, in mediating NR6A1-depleted effects and demonstrated a direct connection between Snail expression and resistance formation. Snail is a transcription factor capable of negative regulation of transcription by binding E-box sequences. Among its major targets is E-cadherin, a component of cell contacts responsible for epithelial features and acting as an onco-suppressor^[27]^. Snail is upregulated by various signaling pathways, including mitogen-activated protein kinases, receptor tyrosine kinases, the NF-κB pathway (inflammation), hypoxia, Notch (related to embryonic development), and others^[28]^. Snail stimulates invasiveness and migration of cancer cells by suppressing E-cadherin, activating the expression of integrins and N-cadherin, and influencing the composition of basement membrane proteins (suppression of laminins)^[29]^. Metastasis triggered by EMT is a leading reason for cancer-associated mortality^[30]^. Therefore, the involvement of Snail, an EMT regulator, in the development of hormonal resistance through the NR6A1/DNMT3A axis is of particular interest. Additional research is needed to estimate the frequency of NR6A1/DNMT3A inhibition among resistant cancer cells and to delineate the perspective of using the current parameters as an adjunctive prognostic criterion for tumor resistance.

## METHODS

### Cell lines and assessment of antiproliferative activity

The MCF-7 (ATCC HTB-22) and MDA-MB-231 (ATCC HTB-26) cell lines were cultured at 37 °C in 5% CO_2_ in DMEM medium (PanEco, Russia), supplemented with 4.5 g/L glucose and 10% fetal bovine serum (HyClone, USA). To establish drug-resistant sublines, parental MCF-7 cells were exposed to rapamycin or tamoxifen (Cayman Chemical) over a prolonged period, yielding the MCF-7/Rap and MCF-7/T sublines, respectively^[16,31]^. Experiments on resistant cells were conducted at least two months post-drug withdrawal. Cell viability following rapamycin or tamoxifen treatment was assessed using modified MTT assays as described in^[32,33]^.

### Transient transfection and reporter gene activity assays

To evaluate transcriptional activities of estrogen receptor alpha (ERα) and Snail, cells were transiently transfected with luciferase reporter plasmids containing estrogen-responsive elements (ERE-Luc) or Snail-sensitive E-cadherin promoter sequences, following the method in^[34]^. A β-galactosidase plasmid was cotransfected to monitor transfection efficiency, with the luciferase/β-galactosidase activity ratio calculated as relative luciferase activity. For BRCA1 promoter activity analysis, cells were cotransfected with a luciferase vector driven by the BRCA1 promoter and a β-galactosidase plasmid; the BRCA1-luc construct was described by Bindra *et al.* 2005^[35]^. To express wild-type Snail (wtSnail), transfections were conducted using pcDNA3-Snail-HA or the corresponding empty vector. All transfections employed Lipofectamine 2000 (Thermo Fisher Scientific, USA). The pcDNA3-Snail-HA plasmid was described by Dave *et al*. 2011^[36]^, and E-cadherin promoter luciferase reporter plasmid was described by Reid *et al.* 2003^[37]^.

### DNA constructs

The siRNAs most effective in suppressing *NR6A1* expression in the MCF-7 cell line in preliminary experiments were used for cloning into the lentiviral vector pLKO.1-TRC. The target sequence of the *NR6A1* mRNA (NM_033334.4) corresponding to position 850-869 5’-CCTCCTCCACACATTACCAATATATAT-3’ was then integrated into a hairpin structure and cloned into the pLKO.1-TRC lentiviral vector between the AgeI and EcoRI restriction sites. Insertion sequences confirmed by Sanger sequencing (Evrogen, Russia).

### Infection and selection of infected cell cultures

To obtain lentiviral particles, the following plasmids were cotransfected into HEK293FT (R70007, Thermo Fisher Scientific, USA) packaging cells: pLKO.1-TRC (#10878, Addgene, USA) encoding target shRNAs and auxiliary packaging plasmids pΔR8.2 (#12263, Addgene, USA), pVSV-G (#8454, Addgene, USA). GenJect-39TM transfection reagent (Molecta, Russia) was used for transfection in accordance with the manufacturer's instructions. To exclude the off-target effects of *NR6A1* shRNAs, we used the parallel infection with virion particles formed by the same lentiviral vector-coding shGFP (pLKO.1 GFP shRNA #30323, Addgene, target sequence: 5’GCAAGCTGACCCTGAAGTTCAT3’), which is absent in the human genome. The viral particles contained in the conditioned medium were collected 24 and 48 h after transfection and then added to MCF-7 cells in the presence of 8 µg/mL Polybrene (Sigma, USA). Infected MCF-7 cells were then selected using puromycin (Sigma, USA) at a concentration of 1 µg/mL for 4-5 days.

### Total RNA isolation and quantitative real-time PCR

Total RNA was isolated from cells using TRIzol reagent (Invitrogen, USA) according to the manufacturer’s protocol. The cDNA was prepared from 1 μg of total RNA by reverse transcription using the iScript™ Advanced cDNA Synthesis Kit (Bio-Rad, USA). Real-time quantitative PCR was performed using 5X qPCRmix-HS SYBR (Evrogen, Russia) on the CFX96 Touch Real-Time PCR Detection System (Bio-Rad, USA) in accordance with protocol: initial denaturation for 3 min at 95 °C, followed by 40 cycles at 95 °C for 15 s, at the annealing temperature (Ta, °C), depending on the target gene [Table 1], and at 72 °C for 30 s. All PCR reactions were conducted in three technical repeats with beta-actin (ACTB) as a normalizer. Primers used for RT-PCR are listed in Table 1. Relative mRNA expression was determined using the ΔΔCt method^[38]^.

**Table 1:** Primers used for RT-PCR

**Gene**	**Forward primer** **(5’-3’)**	**Reverse primer** **(5’-3’)**	**T annealing, °C**
*NR6A1*	AGAGCTTGACCCAGGCACTA	GCTTGAAAAACCCTTTGCAG	58
*DNMT3a*	TGGCAGGATAGCCAAGTTCAG	GCTGGTCTTTGCCCTGCTTTATG	60
*ACTB*	ATGTGGCCGAGGACTTTGATT	AGTGGGGTGGCTTTTAGGATG	60

### Immunoblotting

The cell lysates were prepared for immunoblotting as reported earlier in^[39]^. The lysates were separated on 10% SDS-PAGE gels, transferred onto a nitrocellulose membrane (Amersham), and treated as reported previously^[40]^. To monitor the transfer efficiency, proteins were visualized by briefly (90 s) incubating the membranes in Ponceau S dye solution^[41,42]^. After treatment in 5% nonfat milk (AppliChem), the membranes were processed with primary antibodies (Cell Signaling Technology) at 4 °C overnight. The antibodies directed against α-tubulin (Cell Signaling Technology) were applied for the standardization of loading. The secondary antibodies matching IgGs conjugated to horseradish peroxidase were purchased from Jackson ImmunoResearch. The protocol of Mruk and Cheng^[43]^ was applied to conduct the detection using the ImageQuant LAS4000 chemiluminescence system (GE HealthCare). Densitometry analysis for immunoblotting data was performed using ImageJ software (Wayne Rasband). The protocol for densitometry was provided by the University of Queensland with the recommendations from the works^[44]^.

### Bisulfite pyrosequencing for methylation analysis

Methylation analysis was performed using bisulfite pyrosequencing. The ExtractDNA Blood & Cells Kit (Evrogen, Russia) was used to isolate genomic DNA from the cell lines. EZ DNA Methylation-Gold™ Kit (Zymo Research, USA) was used for DNA bisulfite processing. All conversion steps were performed according to the manufacturer's protocol. For amplification of bisulfite-treated DNA, we used a ready mix for PCR 5X MaSTaqDD (Dialat Ltd., Russia) with the addition of gene-specific primers listed in Table 2. The PCR products were sequenced with the specific sequencing primers as indicated in Table 2 by PyroMark Q24 (Qiagen, Germany). The methylation level of the CpG sites of the studied sequences was evaluated using the PyroMark Q24 Advanced software.

**Table 2:** The primers and assays used for pyrosequencing

**Gene**	**Forward primer** **(5’-3’)**	**Reverse primer** **(5’-3’)**	**Sequencing primer** **(5’-3’)**	**Assay**
*LINE-1*	TGAGTTAGGTG TGGGATATAGT	bio~AAAATCAAA AAATTCCCTTTC	GTTAGGTGTGG GATATAGTTT	YGTGGTGYGTYGTTTTT TAAGTYGGTTTGAAAAG YGTAATATTYGGGTGGGA
*NR6A1 cg09538139*	bio~AGTATTTTTT GTAATGTAGGTTTTT	ATTTTATTTATATCT ATTTAATATTTTCCT	ATATCTATTTA ATATTTTCCTCC	TCRATATTTACCR CRAAATATTTTTA
*NR6A1 cg14590644*	bio~TCCCTCCA AAACCTATCCTAC	GTTGTATTTTTGT AAGATTAGTGTG	GGTAAGGAATG ATTTGTTTTAG	GYGTTTTTTTT AGGTTAATTAT AGYGTTTTGGGT
*NR6A1 cg14046477*	TTTAATTTGAAATTT GTTATTTGTTAGATA	bio~AAAAACCTACA TTACAAAAAATACTC	GGTTTTAGATT TTTTAGAGAATT	GYGAGTTTATGGYGTAGYGA GGGGAGGAGGTATTGATTT
*DNMT3A cg03463641*	TAGTATTGGGGT TGGGGATAGTAG	bio~ACCTTAA CCCTATAAAAC AAAATAACCTC	TAGTATTGGGGT TGGGGATAGTAG	YGTTGGTTTAAT YGYGTYGTAATT TTTAG
*DNMT3A cg21629895*	TGGAAGATTTTGT GTGTGTTTATATAT	biotin-AAATCAAAA ACCTAAAACCCTAAAC	TGGAAGATTTTGTG TGTGTTTATATAT	YGTTTTTATTTTTTTAT YGTGGGGGTTGTTTTTT TTTTTTATGGAGYGTTT

### Statistical and bioinformatics analysis

Each antiproliferative assay was repeated three times. Microsoft Excel and GraphPad were used to perform the statistical analysis. Results were reported as mean ± S.D. (standard deviation value) unless specifically indicated. A *P*-value < 0.05 was regarded as statistically relevant. *T*-test was applied for the comparison of means. Correlation analysis using Pearson and Spearman coefficients between gene expression and methylation in a breast cancer sample panel from the TCGA (BRCA-TCGA) was performed with the SMART (Shiny Methylation Analysis Resource Tool) application (www.bioinfo‑zs.com/smartapp/)^[45]^.

## RESULTS

### Influence of tamoxifen and rapamycin on MCF-7 cells

**Figure 1:**
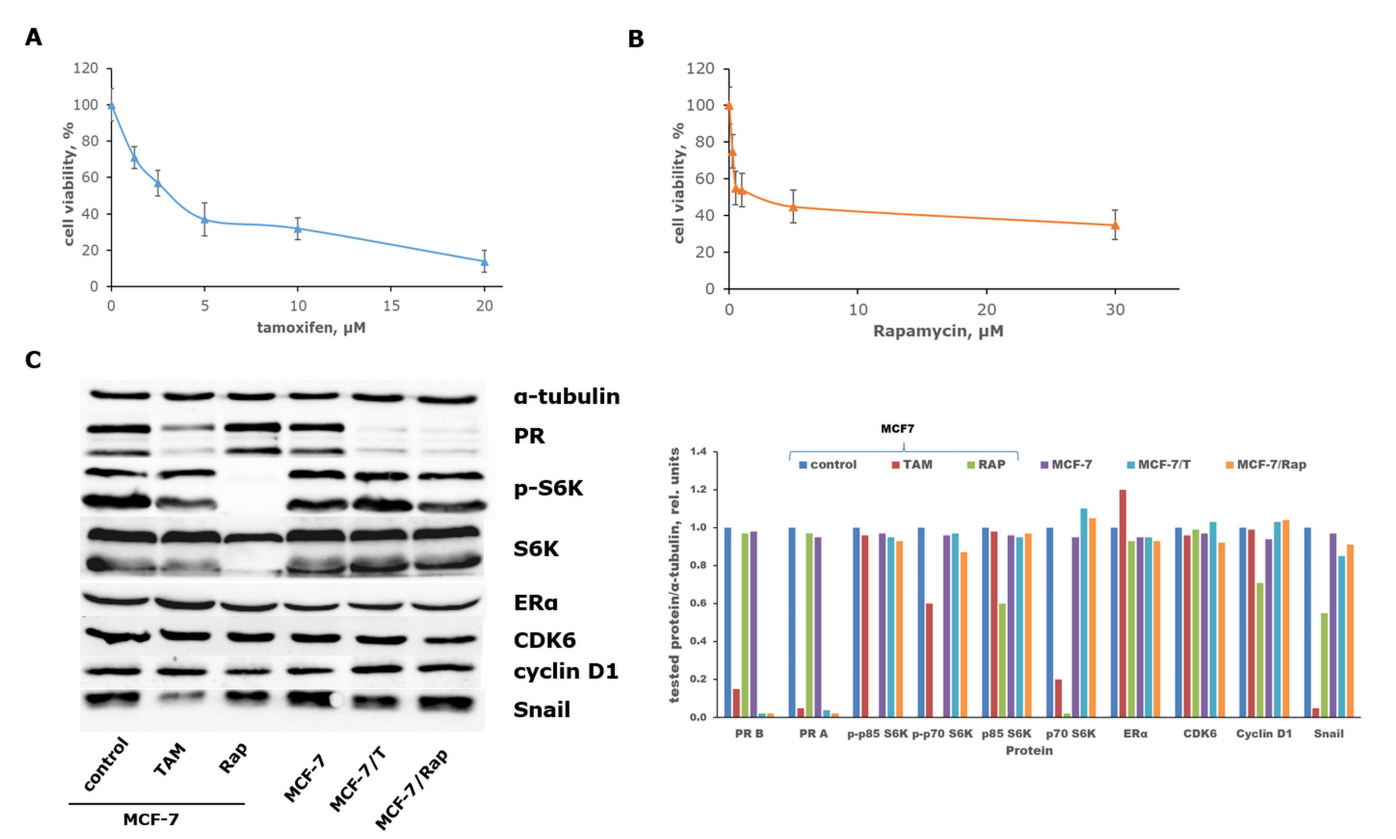
MCF-7 cell line sensitivity to antiestrogen tamoxifen (A) and mTOR inhibitor rapamycin (B). MCF-7 cell line was exposed to the mentioned compounds within 72 h, and the cell survival was measured by the MTT assay. The results represent the mean value ± S.D. from three independent experiments. (C) Western blotting of the MCF-7 protein samples exposed to 5 μM tamoxifen or 0.5 μM rapamycin within 24 h and protein samples of the MCF-7 cells and MCF-7/T and MCF-7/Rap sublines. PR - progesterone receptor, p-S6K - phosphorylated ribosomal protein S6 kinase, S6K - ribosomal protein S6 kinase, ERα - estrogen receptor α, CDK6 - cyclin-dependent kinase 6. To monitor the transfer quality, samples were visualized by incubating the membranes in Ponceau S dye solution^[41,42]^. The α-tubulin antibodies were used as loading control. Densitometry for the tested proteins/α-tubulin ratio was carried out using ImageJ software (version 1.53m). One of two replicates is shown.

Short-term exposure of MCF-7 cells to two types of anticancer drugs, selective ERα modulator tamoxifen [Figure 1A] and mTOR inhibitor rapamycin [Figure 1B], resulted in a significant reduction in cell growth. A parallel analysis of key signaling proteins confirmed distinct mechanisms of action for each drug. Tamoxifen treatment notably suppressed estrogen-responsive genes (PR, progesterone receptor), whereas rapamycin significantly inhibited mTOR-dependent S6 kinase. Furthermore, rapamycin’s effects were associated with a negligible decline in cyclin D1 expression, no change in CDK6 levels, and suppression of the epithelial-mesenchymal transition protein Snail. Tamoxifen also demonstrated anti-Snail potency [Figure 1C, left part].

**Figure 2:**
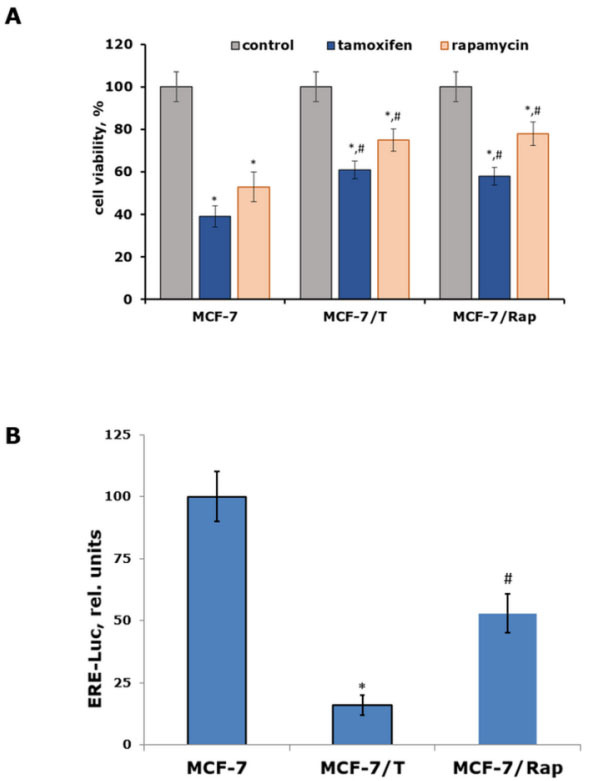
(A) The cross-resistance of MCF-7, MCF-7/T, and MCF-7/Rap cell sublines to tamoxifen and rapamycin. The cells were processed with the mentioned derivatives within 72 h, and the cell survival was measured by the MTT assay. The data reflect the mean value ± S.D. from three independent experiments, ^*^P < 0.05 - compared to respective control cells, ^#^*P <* 0.05 - *vs.* MCF-7 cells treated with respective drug (*t*-test was applied); (B) Reporter assay of the transcriptional activity of ERα. The relative luciferase activity (ERE-Luc) was measured in arbitrary units as the relation of luciferase to β-galactosidase activity. The data are the mean ± S.D. from three independent trials, ^*^P < 0.05 - in comparison with MCF-7 and MCF-7/Rap cells, ^#^*P <* 0.05 - *vs.* MCF-7 and MCF-7/T cells.

The next experiments were performed on the tamoxifen-resistant (MCF-7/T) and rapamycin-resistant (MCF-7/Rap) sublines selected by long-term exposure of the parental cells to the respective drugs. Despite the different mechanisms of selected drug actions, both resistant sublines are described as cross-resistant to rapamycin and tamoxifen^[16,31]^. The control experiments confirmed the retention of increased resistance to both drugs in the MCF-7/T and MCF-7/Rap cell sublines [Figure 2A]. Examination of the resistant cell protein profile showed high expression of mitogen proteins and a partially restored level of Snail expression, while the expression of estrogen-dependent progesterone receptor protein was irreversibly inhibited in both resistant lines [Figure 1C, right part]. The suppression of estrogen receptor α signaling was confirmed by the reporter assay of the ERα transcriptional activity, demonstrating the significant inhibition of that in the resistant sublines [Figure 2B]. As shown, the suppression of estrogen signaling was not associated with the lack of ER. We suggest that this effect may be caused by the disorders of epigenetic regulation of ER expression, particularly with the change in the balance between ER co-regulators and repressors. Totally, the revealed features of cell signaling, namely the suppression of estrogen signaling associated with the reactivation of mitogenic proteins, might be regarded as a key determinant of the influence of the cell cross-resistance on different inhibitors, such as tamoxifen or rapamycin.

### NR6A1 and tamoxifen/rapamycin resistance. NR6A1 and DNA methylation

**Figure 3:**
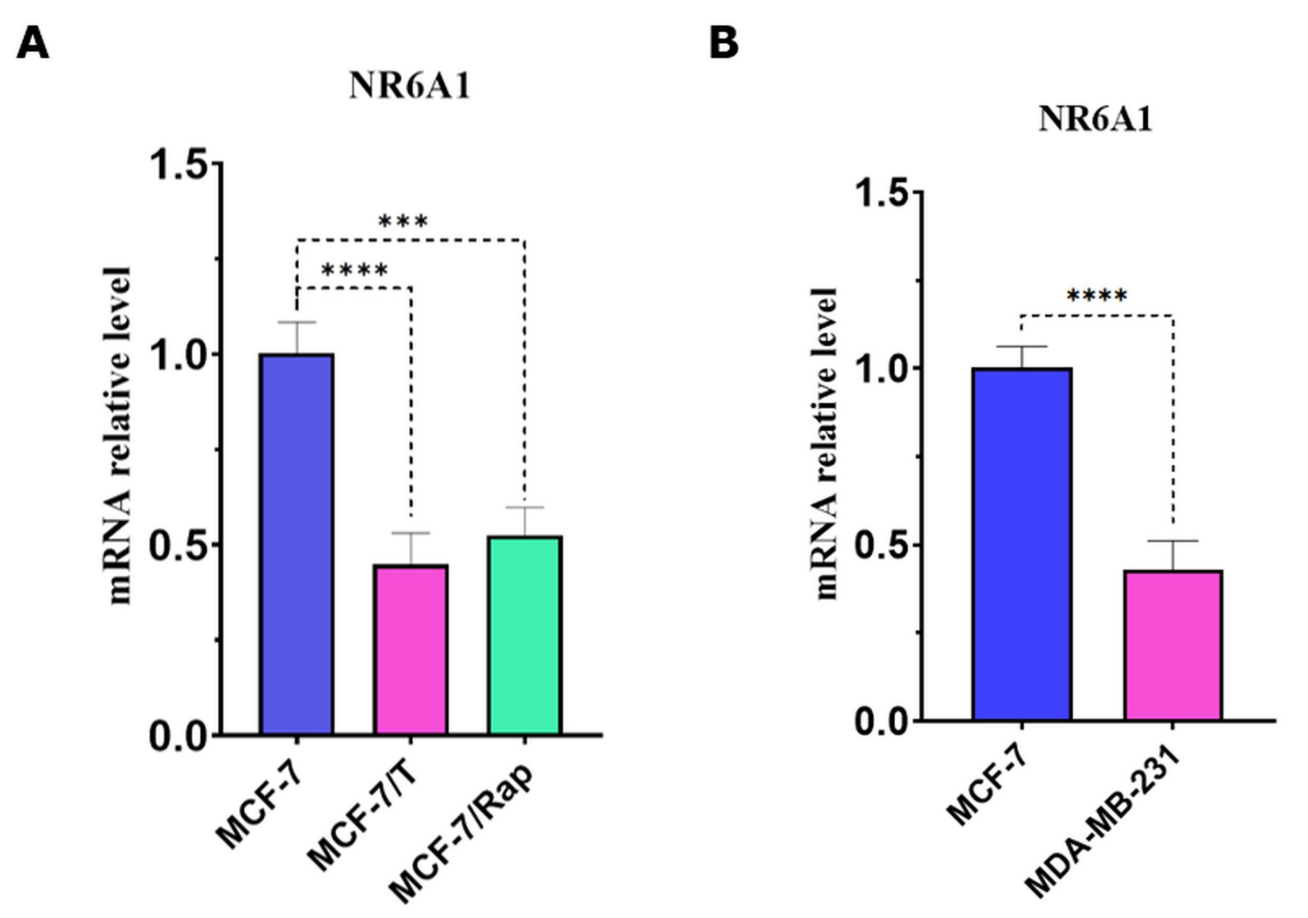
RT-PCR analysis of the *NR6A1* expression in ERα-positive MCF-7, MCF-7/T, and MCF-7/Rap cells (A), and ERα-negative MDA-MB-231 cell line (B); ^****^P < 0.0001, ^***^P < 0.001 (*t*-test was used to compare two sample means).

Earlier, we have shown that the resistance of breast cancer cells to rapamycin or tamoxifen is related to decreased DNMT3A expression^[16,17]^. Here, we have analyzed the expression of *NR6A1*, which is now considered one of the possible transcriptional factors involved in the regulation of DNA methyltransferases and, based on recent publications, may be linked to the modulation of hormonal signaling^[20,21,25,26,46]^. The analysis of mRNA *NR6A1* content in the parent and resistant MCF-7 cells revealed a significant decrease in *NR6A1* expression in both MCF-7 resistant sublines [Figure 3A]. In agreement with that, a comparable reduction in the expression of NR6A1 was detected in the independent resistant cell line, MDA-MB-231 [Figure 3B].

**Figure 4:**
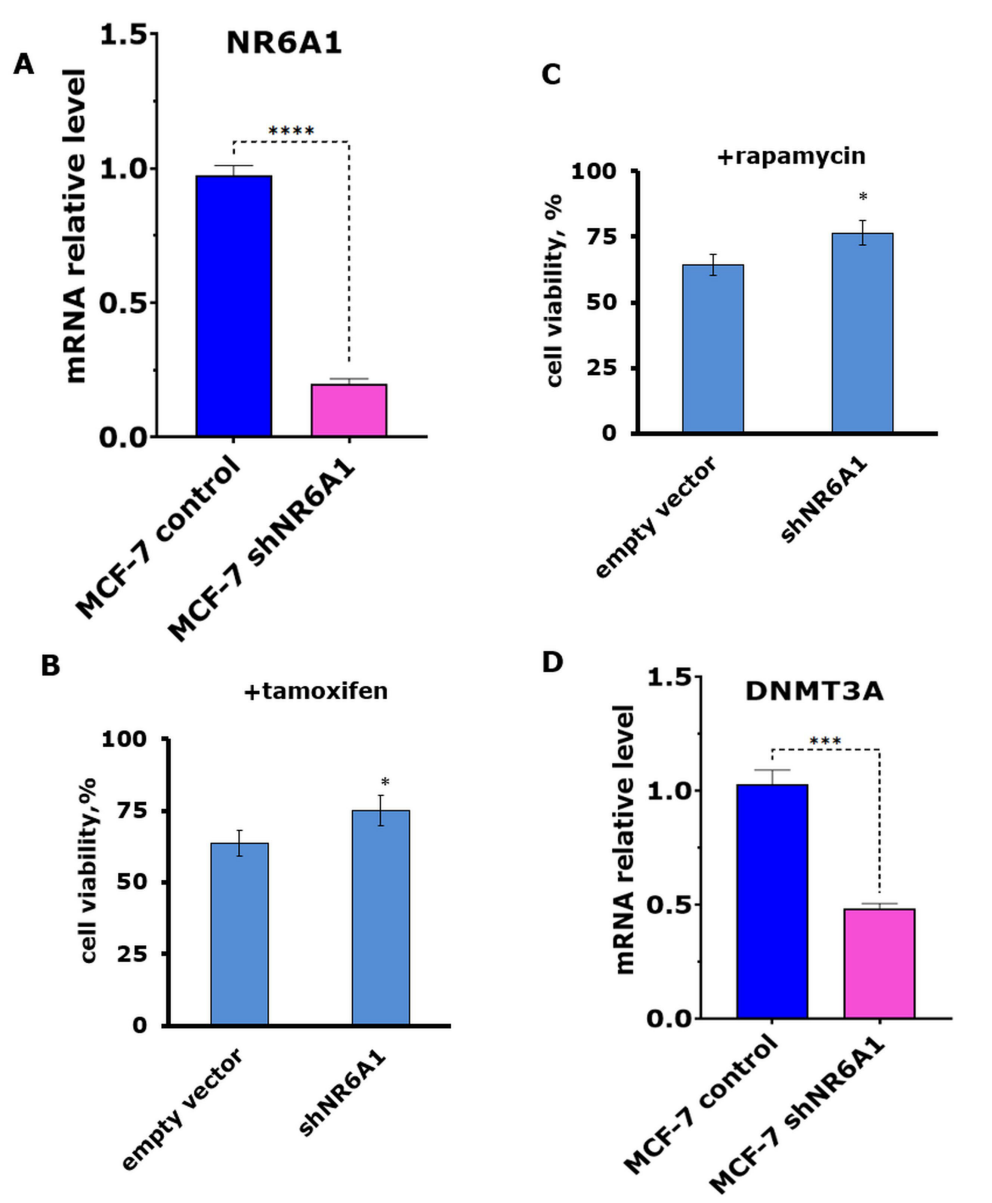
(A) The expression of NR6A1 in the MCF-7 cells after *NR6A1* knockdown; ^****^P < 0.0001. The MCF-7/SCR and MCF-7/shNR6A1 cell line sensitivity to tamoxifen (B) and rapamycin (C); ^*^P < 0.05 - compared to cells exposed to empty vector. The data reflect the mean ± S.D. from three independent trials; (D) RT-PCR analysis of DNMT3A expression in MCF-7 cell line after *NR6A1* knockdown; ^***^P < 0.05.

Knockdown of *NR6A1* in the parent MCF-7 cell line [Figure 4A] caused a partial reduction in the cell sensitivity to tamoxifen and rapamycin, supporting the participation of NR6A1 in resistance development [Figure 4B and C]. Importantly, analysis of the DNMT3A level revealed a decrease in DNMT3A expression in the NR6A1 knockdown cells [Figure 4D], showing the interrelationship between NR6A1 and DNMT3A signaling.

**Figure 5:**
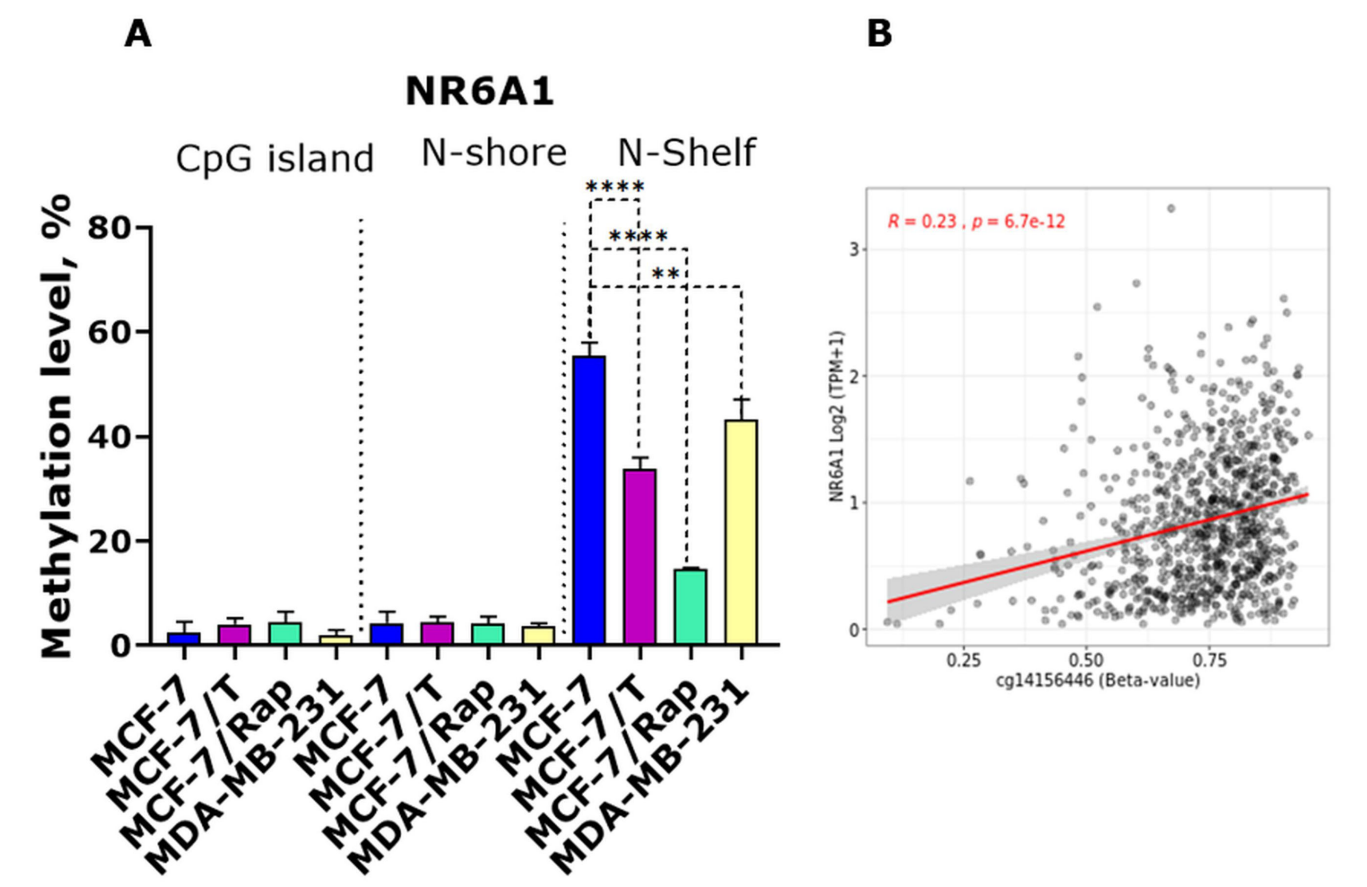
(A) Methylation in the CpG island, N-Shore, and N-Shelf sequences at the *NR6A1* locus in MCF-7, MCF-7/T, MCF-7/Rap, and MDA-MB-231 cells. ^**^P < 0.01, ^****^P < 0.0001 (*t*-test was used to compare two sample means); (B) Correlation between NR6A1 expression and methylation for cg14156446 (TCGA-BRCA dataset: 853 cases).

To further investigate the role of DNA methylation in the *NR6A1* expression regulation, the study of methylation in the CpG island, N-Shore, and N-Shelf sequences at the *NR6A1* locus was performed. A significant decrease in DNA methylation in the N-Shelf sequence of the *NR6A1* gene (genomic coordinates chr9:127284871-127285071, hg19/Human) was shown both in the MCF-7-resistant derivatives and in the MDA-MB-231 cell line [Figure 5A]. Analysis of the breast invasive carcinoma (TCGA-BRCA) dataset showed that methylation of the cg14156446 region is associated with increased NR6A1 expression [Figure 5B]. Thus, the reduced *NR6A1* gene expression of resistant cells is associated with inhibition of DNMT3, which in turn might maintain the low level of *NR6A1* via hypomethylation in the N-Shelf sequence of the NR6A1 gene (e.g., in the sequence around cg14156446). This proposal is in accordance with our previous findings that demonstrated the low DNMT3a level in the resistant cell sublines^[17]^.

### NR6A1 knockdown and the growth-related protein expression. NR6A1 and Snail regulation

**Figure 6:**
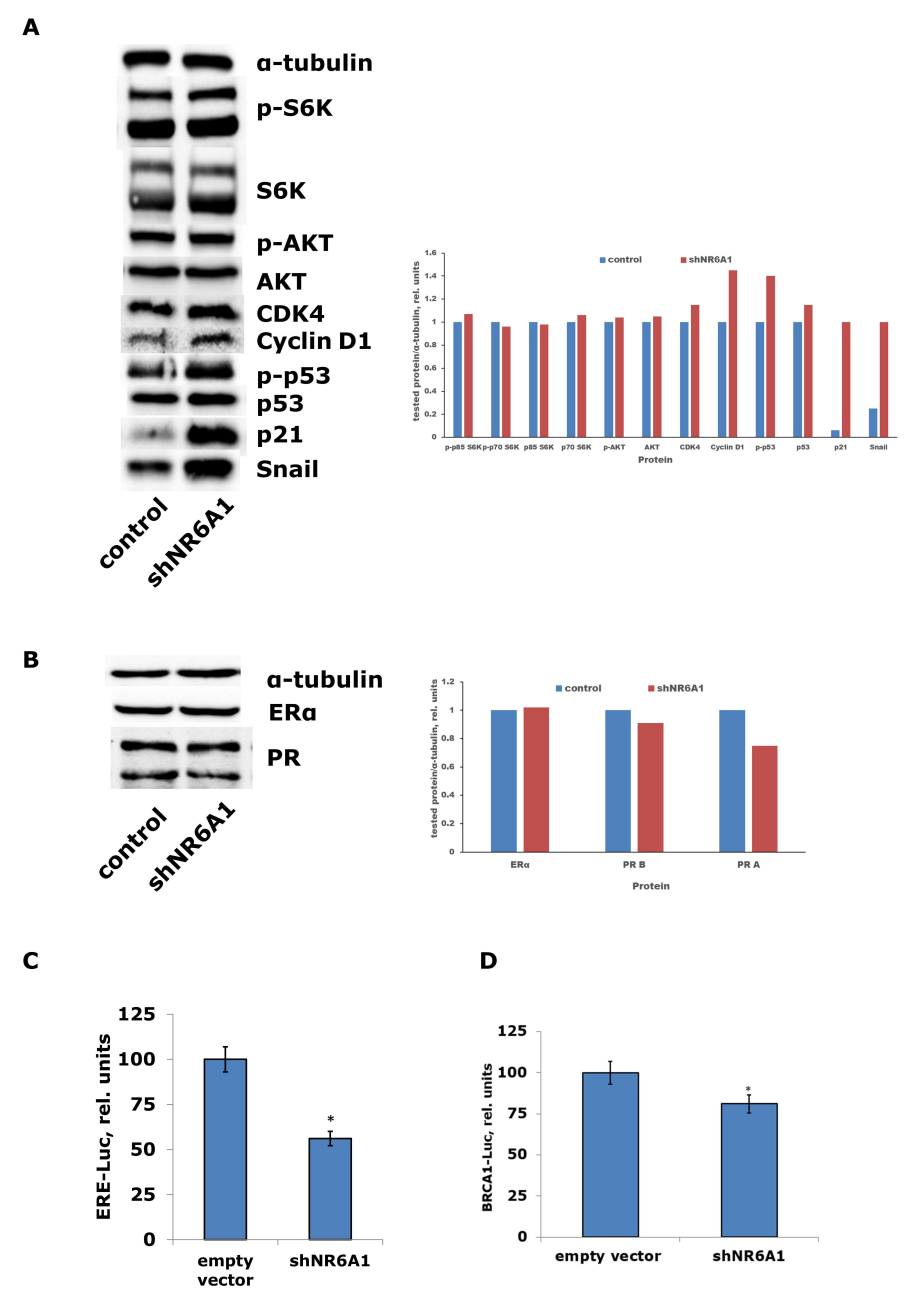
(A) Immunoblotting analysis of the general signaling proteins in MCF-7 cells after *NR6A1* knockdown; (B) Immunoblotting analysis of the estrogen receptor α signaling proteins in MCF-7 cells after *NR6A1* knockdown. Densitometry for the tested proteins/α-tubulin ratio was performed using ImageJ software (version 1.53m); (C) Reporter assay of the ERα-responsive element after *NR6A1* knockdown; (D) Reporter assay of the BRCA1 promoter after *NR6A1* knockdown. The data are the mean ± S.D. from three independent tests; ^*^P < 0.05 - in comparison with cells treated with empty vector (*t*-test).

Western blotting of the signaling proteins in the NR6A1 knockdown cells revealed the activation of the key mitogenic proteins, CDK4 and cyclin D1, and marked activation of Snail, one of the crucial proteins of epithelial-mesenchymal transition, while the expression of other growth-associated proteins was not changed [Figure 6A]. Increased expression of these proteins was accompanied by partial activation of proapoptotic p53/p21 signaling. The study of estrogen receptor α signaling in the NR6A1 knockdown cells showed an unchanged level of ERα protein and slight suppression of PR protein level [Figure 6B], whereas the reporter analysis of ERα transcriptional activity demonstrated the marked suppression of the latter [Figure 6C]. Moreover, analysis of the promoter activity of BRCA1, one of the key estrogen-responsive genes, revealed a partial decrease in activity following *NR6A1* knockdown [Figure 6D], highlighting a weakened estrogen machinery in these cells.

**Figure 7:**
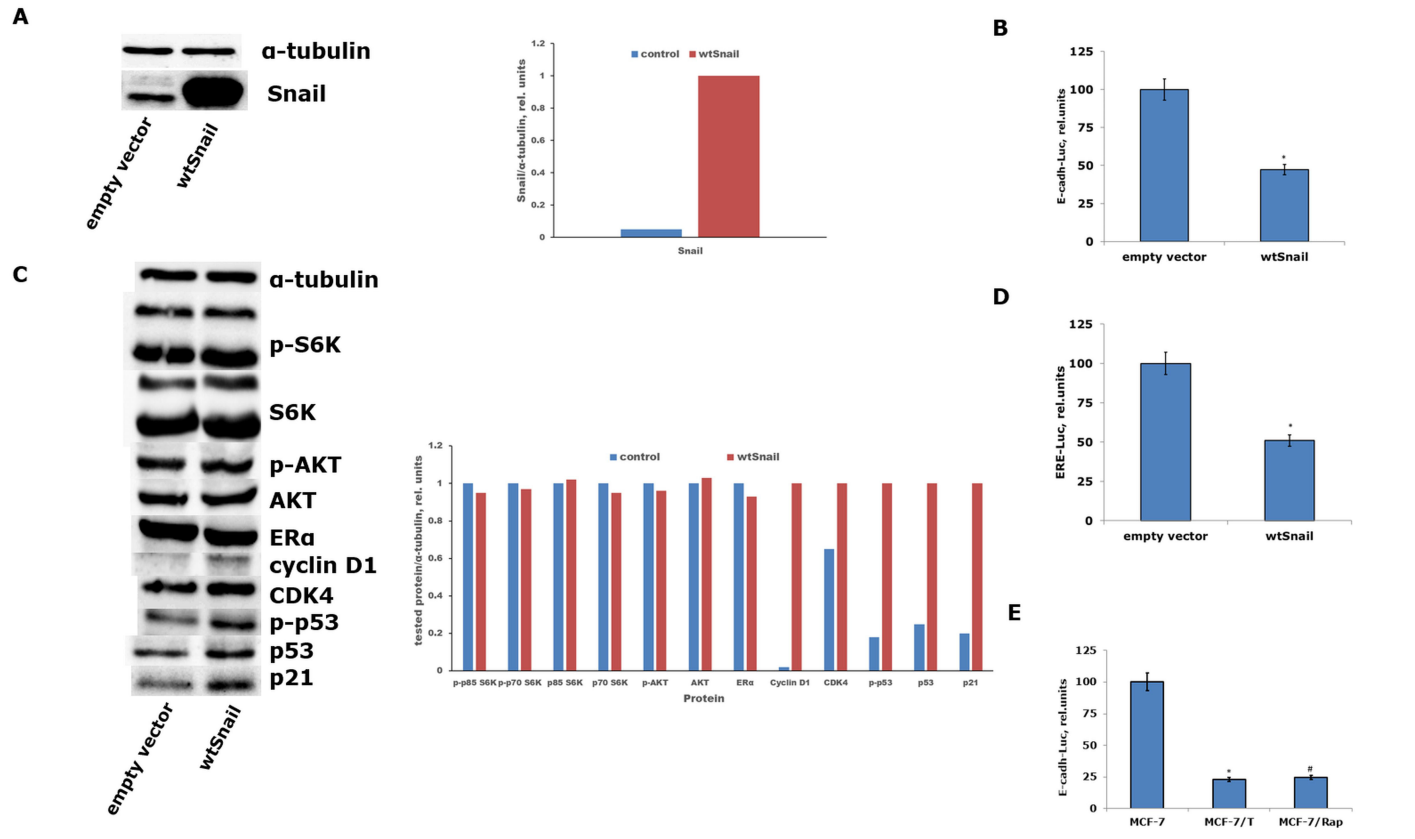
(A) Western blotting assay of Snail expression in MCF-7 cell line after transfection with the wtSnail plasmid. Densitometry for Snail/α-tubulin ratio was carried out using ImageJ software (version 1.53m); (B) Reporter analysis of Snail transcriptional (trans-repressor) activity after wtSnail transfection. wtSnail-transfected MCF-7 cells were subjected to transfection with the luciferase reporter gene construct bearing Snail-sensitive wild-type E-cadherin promoter sequences. The data reflect the mean ± S.D. from three independent assays; ^*^P < 0.05 - in comparison with cells treated with empty vector (t-test was applied); (C) Immunoblotting analysis of the MCF-7 cell protein samples after wtSnail transfection. Densitometry for the indicated proteins/α-tubulin ratio was carried out using ImageJ software (version 1.53m); (D) Reporter assay of the ERα transcriptional activity in Snail-transfected cells. The data are the mean ± S.D. from three independent tests; ^*^P < 0.05 - compared to cells treated with empty vector (*t*-test was used to compare two sample means); (E) Reporter assay of Snail transcriptional (trans-repressor) activity in MCF-7, MCF-7/T, and MCF-7/Rap cell sublines. The data reflect the mean ± S.D. from three independent trials; ^#,*^P < 0.05 - compared to luciferase activity in MCF-7 cells, which is taken as 100 relative units (*t*-test was used to compare two sample means).

Recently, we and others have demonstrated Snail involvement in the stimulation of apoptotic signaling and suppression of estrogen machinery in breast cancer cells^[47,48]^. To further elaborate on the role of Snail in mediating NR6A1 knockdown effects, MCF-7 cells underwent transfection with plasmids expressing the wild-type variant of Snail. The Snail plasmid transfection resulted in a substantial increase in the Snail protein level [Figure 7A], accompanied by an enhancement of Snail transcriptional (trans-repressor) activity [Figure 7B]. A study of the signaling protein expression detected changes in the protein expression in the Snail-transfected cells similar to those in NR6A1 knockdown cells: activation of mitogenic CDK4 and cyclin D1, increased p53/p21 signaling [Figure 7C], and suppression of ERα transcriptional activity [Figure 7D], supporting the pivotal role of Snail in mediating the effects of NR6A1 depletion.

It was indicated above that brief exposure of the MCF-7 cell line to tamoxifen or rapamycin was accompanied by a decrease in Snail expression when the resistant MCF-7/Rap and MCF-7/T cell sublines were characterized by a partially restored level of Snail expression [Figure 1C]. The different Snail expression in the resistant cells, namely, the relatively low Snail level in the MCF-7/T cells, may be caused by the irreversible inhibition of Snail expression under long-term tamoxifen selection. At the same time, reporter analysis of the Snail trans-repressor activity revealed a significant increase in the latter in the resistant cells [Figure 7E], underlining the direct association between the NR6A1/Snail axis and the formation of the cell resistance phenotype.

## DISCUSSION

It is known that resistance to various damaging cytostatic agents is one of the universal characteristics of malignant neoplasms. First of all, it refers to the development of multidrug resistance (MDR), which is caused by the activation of ABC transporters involved in the removal of xenobiotics from the cell. It is multidrug resistance that determines the formation of both primary and acquired resistance to antineoplastic drugs and is one of the key causes of the relatively low efficiency of chemotherapy. The situation is different with tumor resistance to targeted drugs, in the development of which MDR is relatively unimportant. The mechanism of resistance development to targeted drugs is well understood and, in most cases, involves the compensatory activation of unblocked signaling proteins, which restores tumor growth^[49,50]^. However, the molecular mechanism of such rearrangement of signaling pathways is insufficiently studied; the nature of factors responsible for such switching of signaling pathways remains unclear.

The progression of resistance to hormonal drugs is mainly similar to the development of resistance to targeted drugs. Both involve the activation of growth-dependent pathways that bypass blocked hormone-dependent signaling, enabling tumor cells to continue proliferating actively^[51]^. This raises the same questions: What is the origin of the factors contributing to the activation of hormone-independent signaling pathways? Which mechanism is responsible for maintaining resistance-associated changes in growth signaling?

Previously, we developed the MCF-7 cell sublines resistant to hormonal drugs (antiestrogen tamoxifen) and targeted drugs (mTOR inhibitor rapamycin) and demonstrated the effect of cross-resistance of the developed sublines to both drugs. The effect of cross-resistance of the selected cells to tamoxifen and rapamycin may be based on the reactivation of the common growth-related pathways along with irreversible suppression of estrogen signaling. Importantly, recent data showing the effect of partial restoration of tamoxifen sensitivity under blocking of mTOR signaling^[52,53]^ support the importance of growth pathways in the development of the resistant phenotype. Importantly, while the experiments with the resistant cells were performed after two months following drug withdrawal, continued cell cultivation did not drastically decrease the level of drug resistance. The resistant cells were found to be described by a strong DNMT3A suppression; the subsequent experiments with DNMT3A knockdown cells showed the interrelation between DNMT3A suppression and the progression of hormonal/target resistance^[17]^. Currently, to explore the possible mechanism of DNMT3A regulation in resistant cells, the transcriptional factor NR6A1, a relatively recently described protein presumably involved in the regulation of DNA methyltransferases, is being investigated.

NR6A1 is able to link to DNA sequences consisting of a directly repeated (DR0) consensus motif, PuGGTCA^[54]^. NR6A1 partially inhibits the expression of the pluripotency genes Nanog and Oct4^[55]^. In addition, NR6A1 controls the transcription in an indirect manner by competing with transcription activators and regulating miRNAs^[56,57]^.

We found that NR6A1 expression is significantly reduced in drug-resistant cells. Similarly, knockdown of NR6A1 in parental MCF-7 cells suppresses ERα activity, induces partial resistance to rapamycin and tamoxifen, and slightly decreases DNMT3A expression, indicating a functional interplay between NR6A1 and DNMT3A signaling. These findings were further corroborated in independent experiments on the triple-negative, drug-resistant MDA-MB-231 breast cancer cells, which demonstrated a marked reduction in both NR6A1 and DNMT3A expression. Collectively, our data highlight the significant role of the NR6A1/DNMT3A axis suppression in driving the progression of cross-resistance to hormonal and targeted chemotherapies in breast cancer cells.

Our results align with previous studies showing that NR6A1 interacts with and recruits various DNMT isoforms, including DNMT1^[46]^, DNMT3L^[26]^, and DNMT3A^[25]^. Notably, NR6A1 knockdown enhances the phosphorylation of mTOR and AKT, supporting its role in downregulating proliferative signaling pathways^[58]^.

In this study, we identified several signaling proteins negatively regulated by NR6A1, including key mitogenic, proapoptotic, and epithelial-mesenchymal transition (EMT) markers, such as Snail. Previous work, including ours, has shown that Snail suppresses ERα transcriptional activity in breast cancer cells^[47,48]^. Consistent with these observations, we found that Snail overexpression mirrors the effects of NR6A1 knockdown, activating apoptotic pathways and reducing ERα activity. Our data further reveal that drug-resistant cell sublines are characterized by NR6A1 suppression and Snail hyperactivation, suggesting that Snail may serve as a mediator in NR6A1 depletion, potentially contributing to the development of a resistant phenotype.

This study has several limitations. Prior research into NR6A1/DNMT3A signaling in relation to tumor cell resistance remains limited, with only a few studies addressing this association. Further investigation is warranted, particularly *in vitro* and *in vivo* studies that build on this work by examining NR6A1 protein characteristics, including its regulation, folding, and degradation in tumor cells. *Ex vivo* studies of NR6A1 in resistant tumors could also be highly informative.

Overall, our data suggest that the progression of acquired resistance to targeted and hormonal therapies may follow a common mechanism, involving alterations in DNA methylation and shifts in cell signaling pathways to bypass unblocked routes. We propose that coordinated suppression of NR6A1 and DNMT3A may be critical in sustaining the resistant phenotype and could potentially inform the selection of therapeutic regimens for breast cancer in the future.

## DECLARATIONS

### Availability of data and materials

Data can be requested from the authors.

### Copyright

© The Author(s) 2024.
